# Socioeconomic Disparities in the Diversity, Abundance, Structure and Composition of Woody Plants in Residential Streetscapes: Insights for Transitioning to a More Environmentally Just City

**DOI:** 10.3390/plants14243865

**Published:** 2025-12-18

**Authors:** Sandra V. Uribe, Álvaro Valladares-Moreno, Martín A. H. Escobar, Nélida R. Villaseñor

**Affiliations:** 1 Laboratorio de Ecología Urbana (EcoUrban Lab), Departamento de Gestión Forestal y su Medio Ambiente, Facultad de Ciencias Forestales y de la Conservación de la Naturaleza, Universidad de Chile, Av. Santa Rosa 11315, Santiago 8820808, Chile; sandrum@gmail.com (S.V.U.); alvaro.valladares@ug.uchile.cl (Á.V.-M.); marescob@uchile.cl (M.A.H.E.); 2 Grupo de Ecología, Naturaleza y Sociedad, Facultad de Ciencias Forestales y de la Conservación de la Naturaleza, Universidad de Chile, Av. Santa Rosa 11315, Santiago 8820808, Chile

**Keywords:** environmental justice, luxury effect, Santiago de Chile, species richness, socioeconomics, urban greening, urban forest

## Abstract

Vegetation in residential areas plays a crucial role in biodiverse and sustainable cities as it enhances biological diversity, environmental quality, and the human well-being of city residents. However, the distribution of vegetation among these areas is often unequal, leading to disparities in access to its benefits. To promote a more biodiverse and environmentally just city, we investigated how woody plants (trees, shrubs and vines) vary with socioeconomic level in residential streetscapes of Santiago de Chile. Across the city, we sampled woody plants in 120 plots (11 m radius) located in residential streetscapes of three socioeconomic levels: low, medium, and high. A total of 557 woody plants were identified and measured. Of these, only 9.7% corresponded to native species, whereas 90.3% were introduced species. Wealthier residential areas had higher species richness and abundance of woody plants, as well as plants with greater structural size (revealed by height and crown area). In addition, we found that the composition of woody plants differed among socioeconomic levels: *Liquidambar styraciflua*, *Platanus x hispanica*, and *Pittosporum tobira* were more abundant in high socioeconomic areas; *Prunus cerasifera*, *Citrus limon*, and *Ailanthus altissima* were more abundant in medium socioeconomic areas; *Robinia pseudoacacia*, *Acer negundo*, and *Schinus areira* were more abundant in low socioeconomic areas. Our research highlights that woody plant diversity, abundance, structure, and composition vary with socioeconomic level in residential streetscapes. Key insights for reducing these inequalities and achieve a more environmentally just city include: (a) governance and equity-based investment; (b) prioritizing local native species; (c) promoting the use of non-tree woody plants; and (d) empowering communities through capacity building and stewardship.

## 1. Introduction

The relevance of vegetation in urban areas is increasing due to its contribution to human well-being, the environment and biodiversity [1]. Urban vegetation has been identified as an important component of cities that encourages recreational activities, leisure, and sports, helping to improve the physical and mental health of citizens [2,3]. Urban vegetation also contributes to reducing environmental pollution, noise, flooding, and heat islands in highly urbanized cities [4,5,6]. Furthermore, biodiversity in cities is closely related to the presence and diversity of vegetation, which also provides food, rest and refuge for various invertebrate and vertebrate animals [7,8,9].

Vegetation in residential areas is a crucial source of biodiversity in cities, playing a significant role in connecting people with nature [10,11]. Although residential vegetation can produce some disservices such as building damage or even death due to tree or branch fall, the ecosystem services associated with their presence are more valuable and often prevail [2,12]. Therefore, sustainable urban development promotes greener cities, not only considering plant abundance but also plant diversity. The diversity of plants provides urban green spaces with significant resilience to face various threats, such as pests or meteorological events, as some species can resist while others cannot [13,14,15].

In addition to the richness and abundance of trees as “quality” indicators of green spaces, the structural condition of vegetation is an important complementary variable [16,17]. Structural complexity of vegetation in natural ecosystems is associated with resilience and high biodiversity [18]. This complexity is given by several factors. Some of these factors include growth habits, the age of individuals, the vertical and horizontal coverage of plants, and management. In urban ecosystems, vertical cover is also important in cities [19]. For instance, shrubs contribute to noise reduction [20]. Nevertheless, urban vegetation literature commonly focuses on urban trees [21,22,23].

Thus, plants provide a multitude of benefits to urban ecosystems. While climate is typically a primary factor influencing which species thrive in a given natural ecosystem [24], urban environments often feature the same or related ornamental plant species across vastly different global climates [13,25,26]. The introduction of non-native species implies that many are not locally adapted to the climates where they reside, making their survival contingent upon specific human management interventions. For example, sensitive species that require supplemental irrigation to survive after establishment depend heavily on consistent access to water, especially during dry seasons. This management significantly increases the care costs in arid, semi-arid, and Mediterranean climates [27,28].

Therefore, socioeconomic realities can have a close relationship with urban vegetation. In arid regions and those with dry seasons (such as Mediterranean climates), the financial capacity to provide irrigation is not uniform across a city. The income of specific neighborhoods can directly limit the viability and growth of certain sensitive species within urban ecosystems [29,30]. Similarly, the overall abundance of trees and green cover within a neighborhood requires ongoing resources for maintenance (e.g., watering, pruning, pest control). This commonly results in wealthier neighborhoods exhibiting greater plant cover and a higher abundance of trees in residential areas, squares, and parks [31,32].

Despite the importance of vegetation in cities, urban plants and their benefits are not always equally distributed [33,34]. Several countries worldwide have experienced the phenomenon known as the “luxury effect”, where vegetation cover, abundance, and/or tree diversity are greater in zones with higher socioeconomic status [10,35,36]. This effect has been observed in cities of countries with different economic development, but it is more frequent in arid or semi-arid zones and older neighborhoods [36]. However, there are many cases where the luxury effect is not that evident, even in countries with arid climates or dry seasons.

In this research, we aimed to determine whether woody plant diversity, abundance, structure and composition vary with socioeconomic status in residential streetscapes of Santiago de Chile, a city located in a Mediterranean climate. We focused on woody vegetation present in the residential streetscape, which includes trees, shrubs and vines in public spaces (streets, verges, and medians) and in private front yards visible from the street, representing the vegetation that shapes the residential streetscape. Plants in these areas reflect both municipal and resident management, and thus could be closely linked to resident socioeconomic status [37,38]. In particular, we: (1) tested whether the richness and abundance of total, native and introduced woody plants in residential streetscapes vary with neighborhoods’ socioeconomic level; (2) evaluated differences in height and crown area of woody plants, as a structural measure of vegetation, among zones with different socioeconomic levels; and (3) investigated woody plant composition and the species that differed among streetscapes in different socioeconomic areas. Overall, this research provides novel insights into understanding socioeconomic disparities in woody vegetation among residential streetscapes and seeks strategies to promote a biodiverse and environmentally just city.

## 2. Results

### 2.1. Richness and Abundance

We evaluated woody plants in 120 plots located in residential streetscapes of varying socioeconomic levels in Santiago, Chile. Only two of these plots did not contain any woody plants. A total of 108 species were recorded (including morphospecies, Appendix A). We found 45 families and 78 genera (Appendix A), among these, 94 (87%) species were introduced and only 14 (13%) were native (Table 1). The most represented families in terms of number of species were Rosaceae and Fabaceae (12 species each). At the genus level, *Prunus* (6 species) was the most diverse, with all species introduced in Chile (Appendix A). Five introduced species were the most abundant plants, comprising 31.1% of the total: *Robinia pseudoacacia* L. exhibited a relative abundance of 9.0%, *Ligustrum lucidum* W. T. Aiton had a relative abundance of 6.8%, *Liquidambar styraciflua* L. sp. Pl. had a relative abundance of 6.1%, *Acer negundo* had a relative abundance of 4.8%, and *Platanus x hispanica* had a relative abundance of 4.3%. The total species richness recorded in residential streetscapes of low, medium, and high socioeconomic levels were 48, 67, and 60 species, respectively (Table 1).

A total of 557 woody plants were identified and measured. Of the total, 503 individuals (90.3%) corresponded to introduced species and 54 individuals (9.7%) to native species. The mean woody plants per plot were highest in streetscapes of high socioeconomic level with (mean *±* EE) 5.58 (±0.50) woody plants per plot, followed by medium and low socioeconomic levels with 4.56 (±0.44) and 3.9 (±0.39) woody plants per plot, respectively (Table 1).

**Figure 1:**
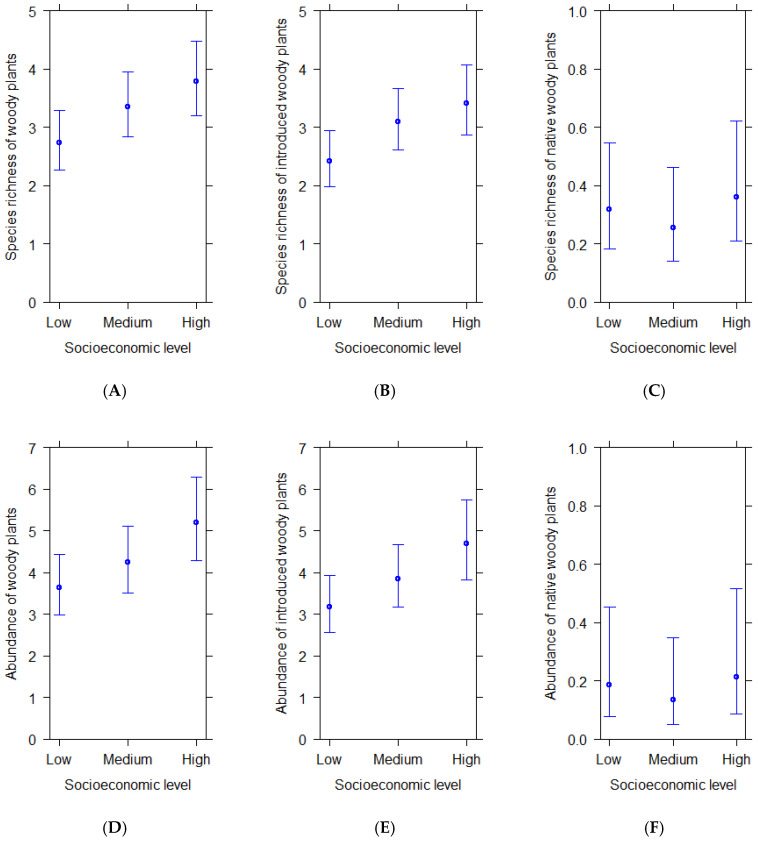
(**A**–**F**) Predicted average species richness and abundance of woody plants per plot in residential areas of different socioeconomic levels according to statistical models. Bars show 95% confidence intervals.

Species richness and abundance of total (Figure 1A,D) and introduced woody plants per plot (Figure 1B,E) increased from zones of low to high socioeconomic level. Significant statistical differences were found between plots from high and low socioeconomic levels in total species richness and total abundance, i.e., streetscapes located in wealthier residential zones had significantly richer and more abundant woody plants than streetscapes located in poorer residential zones (Table 2). Introduced woody plants were also significantly more abundant and richer in streetscapes located in wealthier residential areas than in poor residential areas (Table 2). Native woody plants exhibited similar species richness and abundance in plots across socioeconomic streetscapes (Figure 1C,F; Table 2).

### 2.2. Structure

**Figure 2:**
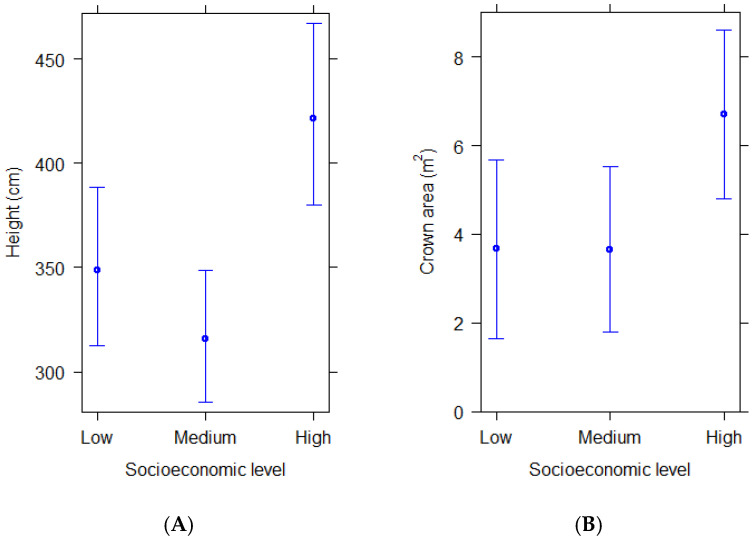
(**A**,**B**) Predicted average height and crown area of woody plants in residential areas of different socioeconomic levels according to statistical models. Bars show 95% confidence intervals.

Structural differences of woody plants also varied with socioeconomics, as revealed by their height and crown horizontal area. Woody plants in residential streetscapes of high socioeconomic level were taller and had larger crown areas than those in residential streetscapes of medium and low socioeconomic level (Figure 2A,B). These differences were significant when comparing both height and crown area between high and medium, and high and low socioeconomic levels (Table 3).

**Figure 3:**
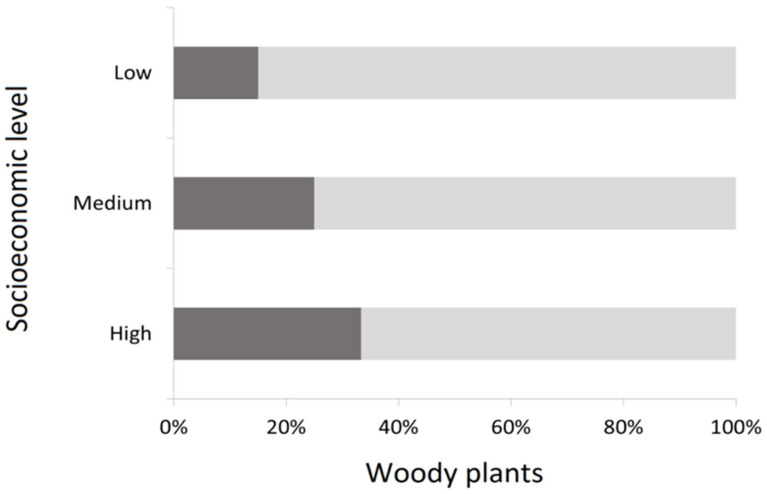
Representativeness of trees (light gray) and non-tree (dark gray) woody plants in streetscapes according to socioeconomic level.

Total species of woody plants are mainly represented by trees (74.9%), with only 25.1% of species represented by other kinds of woody plants such as shrubs *Pyracantha coccinea* M. Roem, *Ligustrum sinense* Lour., and *Pittosporum tobira* (Thunb.) W. Aiton, which are frequently used as hedges. Also, low socioeconomic level zones tend to have a lower proportion of non-tree woody plants than wealthier zones (χ^2^ = 18.51; *p* < 0.05) (Figure 3).

### 2.3. Composition

When comparing common woody species (those present in more than 5% of total plots), permutational multivariate analysis of variance indicated a statistically significant difference in woody plant species composition among residential streetscapes of different socioeconomic levels (*p* = 0.015). SIMPER analysis evidenced different woody species explaining differences among socioeconomic groups (Table 4). *Robinia pseudoacacia* and *L. styraciflua* significantly contributed to the dissimilarity between high versus low socioeconomic areas. *Robinia pseudoacacia* contributed 15% to the total dissimilarity and was more abundant in residential streetscapes of low socioeconomic level than in high socioeconomic level (*p*-value = 0.02). *L. styraciflua* explained 12% of the dissimilarity and was more abundant in residential streetscapes of high socioeconomic level than in the low socioeconomic level (*p* = 0.04). The native species *Schinus areira* L. was more frequent in residential streetscapes of low socioeconomic level than in the high socioeconomic level, although its contribution to dissimilarities was only close to significant (*p* = 0.07, Table 4).

Dissimilarity between high versus medium socioeconomic areas was highly influenced by *L. styraciflua*, which was more abundant in residential streetscapes of high socioeconomic level and emerged as the most significant species, accounting for 13% of the dissimilarity (*p* = 0.001). *Platanus x hispanica* Mill. Ex Münchh. and *P. tobira* also contributed to dissimilarities, being more abundant in residential streetscapes of high socioeconomic level (*p* = 0.03 and *p* = 0.04, respectively).

When comparing plots in residential streetscapes of medium and low socioeconomic level, *Prunus cerasifera* Ehrh., *Citrus limon* (Chritm.) Swingle, and *Ailanthus altissima* (Mill.) Swingle were more abundant in residential streetscapes of medium socioeconomic level than in the low socioeconomic level, significantly contributing to dissimilarity (*p*-value = 0.03, *p*-value = 0.007, and *p*-value = 0.03). *Acer negundo* L. was more abundant in low socioeconomic areas, although it was close to significant (*p* = 0.05).

## 3. Discussion

Vegetation in residential areas is central to urban biodiversity, ecosystem services, and residents’ daily contact with nature. In Santiago de Chile, a highly segregated city located in a Mediterranean climate, residential streetscapes exhibited socioeconomic disparities in woody species richness, abundance, structure, and composition. These differences have implications for the well-being of citizens, adding another barrier for poorer people to access services, specifically ecosystem services and the benefits derived from urban vegetation.

### 3.1. Richness and Abundance of Woody Plants

Woody plant richness and abundance in residential streetscapes increased with the neighborhood socioeconomic level, making evident the occurrence of the “luxury effect” on residential woody plants. This result differed from a previous investigation focused on trees in public parks and squares in Santiago city, where the authors found similar tree abundance and species richness among socioeconomic level zones [39]. In the case of Santiago city, the care of woody plants in residential streetscapes correspond mainly to neighbors as well as 34 different municipalities, whose incomes are highly correlated with their neighbors’ wealth [40], which explains the differences observed in our study. In contrast, vegetation management in parks and squares is commonly managed by municipalities as well as centralized institutions, such as Parquemet [39,41], which has a budget established by the central government that can contribute to reducing disparities in vegetation cover. In addition, the inclusion of non-tree woody plants (shrubs and vines) along with trees in our work could also explain the difference with this previous study, which only evaluated trees in parks and squares [39]. In fact, a recent study found a lack of socioeconomic effects on urban flora sampled in sidewalks, parks, and vacant lots of Santiago, although shrub richness exhibited a luxury effect [42]. Given that different variables can influence the richness and abundance of urban woody plants (e.g., [42]), future studies should explore multiple factors to better understand plant patterns.

The lower species richness and abundance found in streetscapes of low socioeconomic level compared to wealthier zones demonstrate that inequities are far more than purchasing power; it is also a disadvantage in the possibility of enjoying urban plants and their ecosystem services, including a more biodiverse and healthier environment that improves mental and physical health [2,33]. Housing in low socioeconomic zones is characterized by its small size, and then, scarce space to have gardens [43,44]. In some cases, urban planning is so deficient that neither of the streets has land available to plant, and thus, residential streetscapes are dominated by grey structures, including impervious surfaces and buildings. On the other hand, high socioeconomic areas have a larger proportion of green spaces, and residential streetscapes often feature green cover with a greater variety of plants, even though urbanization intensity is high [45].

### 3.2. Structure of Woody Plants

Residential streetscapes in high socioeconomic zones had woody plants with greater height and crown horizontal area than in medium and low socioeconomic zones. This finding can be explained by the varying quality of vegetation management within Santiago city. A recent study showed a substantial inequity in the quality of vegetation pruning across different socioeconomic levels [38]. These authors determined that trees located in residential zones of high socioeconomic level exhibit better pruning quality than trees located in medium and low socioeconomic zones. In this sense, although the objective of corrective pruning—the dominant technique in the management of woody vegetation in the city of Santiago [38]—is to maintain or improve its structure and health, the indiscriminate cutting of branches can heavily reduce tree size through crown reduction [46].

Alternatively, the greater height and crown horizontal area of woody plants found in residential streetscapes of high socioeconomic level could be due to older individuals, since woody vegetation size is a good proxy for woody vegetation age [47]. While the oldest plants can be found in older neighborhoods regardless of socioeconomic status [48], we found that in Santiago, higher socioeconomic areas often invest greater resources to save and prolong tree life. For example, during road infrastructure remodeling in high socioeconomic zones in Santiago city, trees are commonly rescued, maintained and replanted once construction works end (e.g., [49]). Meanwhile, in areas of lower socioeconomic status, vegetation is typically removed and replaced with younger plants after the completion of road infrastructure remodeling or real estate projects.

Although trees are highly represented in streetscapes, shrubs and other non-woody plants play an important role in cities, contributing to heterogeneity and increasing biodiversity and resilience [50]. Nevertheless, in our study, we found that the proportion of non-tree woody plants decreased as the socioeconomic level decreased, adding a new inequity variable to consider in the city. Next, the ecosystem services given by non-tree woody plants, such as noise reduction [20], were more limited in lower socioeconomic conditions. Ecosystem services such as carbon sequestration, thermal regulation or scenic beauty in urban spaces are frequently associated with the presence of trees [51,52,53]. Nevertheless, shrubs and other woody plants, as climbers, can also accomplish these roles, making the mix of different vertical strata complementary and beneficial for urban ecosystems [17,54,55].

### 3.3. Composition of Woody Plants

Our study revealed the predominance of introduced woody plants in residential streetscapes, accounting for 87% of the recorded species, while native species comprise only 13%. These values coincide with the results of [56], who found 88.3% and 11.7% for introduced and native woody species, respectively, on the streets of Santiago city. The reasons for the dominance of introduced woody plant species in residential areas may be diverse. On the one hand, they could be reflecting the European influence on the use of ornamental flora species in urban spaces, which has been present in Chile since the nineteenth century [57]. It could also be due to urban foresters, landscape architects, and residents commonly selecting introduced plants with ornamental value or fast growth for afforestation [41]. On the other hand, the low use of native woody species in residential areas could be due to the lack of knowledge about their use as ornamental plants and the small number of native species commercially cultivated, as well as the misconception that all native Chilean plants grow slowly [56].

Although introduced woody species represent almost 90% of the total plant species, we found differences in woody plant communities in residential streetscapes of different socioeconomic status. In the case of high vs. low socioeconomic levels, the dissimilarity is explained by the high abundance of *R. pseudoacacia* in the low level and of *L. styraciflua* in the high level. Although both species are widely used as ornamental species [58], this difference could be explained by the different budgets available to municipalities for street vegetation management [59]. For example, the commercial value of a *L. styraciflua* seedling is 10 to 15 times higher than a *R. pseudoacacia* seedling. We also found a greater abundance of the native species *S. areira* in residential streetscapes of low socioeconomic level. The greater abundance of this species could be explained by its low commercial value (similar to that of *R. pseudoacacia*) and high drought resistance [58], an attractive condition for municipalities with limited budgets, as it involves less irrigation costs for its maintenance.

*L. styraciflua*, *Platanus x hispanica*, and *Pittosporum tobira* are more abundant in residential streetscapes of high than medium socioeconomic zones. In the case of *L. styraciflua*, its high commercial value limits its use in residential areas of low socioeconomic level. However, *P. x hispanica* and *P. tobira* could be related to their growth characteristics. Although *P. x hispanica* has been widely used for ornamental purposes in the city of Santiago since the beginning of the last century [60], its extensive growth in height and leafy canopy makes it common on sidewalks of large avenues or wide streets [58]. This implies a need for space for its growth, a limited resource in residential areas of lower socioeconomic levels due to the planning of narrow streets to accommodate higher density of buildings and housing construction [61]. Similarly, the bushy growth of *P. tobira* would also require space to grow, a condition that restricts its use only to wide streetscapes common in higher-income areas.

*P. cerasifera*, *C. limon*, and *A. altissima* are more abundant in residential streetscapes of middle than low socioeconomic levels. In Chile, in the late 1970s and early 1980s, a process of suburban expansion of the city intensified, linked to the residential aspirations of the middle class [62]. Thus, residential areas were influenced by North American design with single-story houses and some innovative features for the time, such as the presence of front yards [63] that added aesthetic value to the streetscape. Thus, householders began to plant fruit trees in their front gardens, including *P. cerasifera* and *C. limon*, contributing to the self-sufficiency of these fruits in cities [64]. Additionally, the compact growth dimensions of *P. cerasifera* make it a common species within the vegetation of streets and passages within villas or condominiums [58].

*Acer negundo* was more abundant in low-income areas. The low commercial value of this species (25 to 30 times less than *L. styraciflua* and a third of the value of *R. pseudoacacia* and *S. areira*) has probably encouraged its use in street vegetation by low-income municipalities and by real estate developers of social housing. *Acer negundo* is the fourth most abundant species found in our survey, which could explain the rapid expansion of the introduced maple bug (*Boisea trivittata*) in the city of Santiago since its first record in 2020 [65], helped by the massive use of *A. negundo* in low socioeconomic level municipalities that dominate the urban landscape.

The massive use of the same introduced species in cities with very different climatic and edaphic conditions is a phenomenon that causes biotic homogenization worldwide. Therefore, to conserve biodiversity, we need to promote the use of a variety of native plant species [25,66]. The importance of plant origins in urban ecosystems is currently increasing due to climate change. The requirements of plants to survive in a city must adjust to the new conditions that the climate imposes [67]. In this context, native species better address this challenge [68], especially in our Mediterranean-climate region. Nevertheless, introduced woody plants from temperate climates dominate the current urban forest, and native species represent a very low percentage of residential woody plants in Santiago. In fact, compared to [32], who found 14.0% of native trees at the city-level, our study showed that the percentage of native woody plants was even lower (9.7%) in residential streetscapes. In parks and squares, the proportion of native trees reached 30% [39], still a very low value that raises concerns given the irrigation cost of introduced plants from temperate climates. Given the expected reductions in rainfall in our region due to climate change [69], the challenge is to develop strategies that enhance vegetation resilience to new and future climate conditions. This means that in the future, residential plants must be capable of resisting less or no irrigation. Several Mediterranean native woody plants exhibit this adaptation and possess ornamental potential [70], underscoring the need to incorporate them more extensively into the urban forest.

### 3.4. Recommendations

Our analysis revealed significant disparities in the diversity, abundance, structure, and composition of woody plants across residential streetscapes of varying socioeconomic status in Santiago de Chile. These inequalities contribute to an uneven distribution of the ecological and social benefits provided by urban greenery. To address this environmental injustice and improve the resilience and performance of the city’s urban forest, we propose the following integrated recommendations:

1. Governance and equity-based investment: A centralized, city-wide authority for public vegetation management should be established. This body would be responsible for allocating resources to correct budgetary inequalities between municipalities, ensuring that low-income neighborhoods receive equitable investment. It would also develop and enforce standardized guidelines for woody plant planting, maintenance, and conservation as a matter of state policy. A key function would be to institutionalize a proactive, city-wide tree protection and compensation policy. This policy must prioritize preventing damage to significant trees—such as heritage, large-canopy, or ecologically and socially valuable specimens—during planning and construction. For trees that cannot be preserved in situ, their rescue and replanting should be analyzed, considering prioritizing greening projects in historically underserved areas.
2. Prioritize local native species: To future-proof Santiago’s urban forest against escalating climatic pressures like drought, heat waves, and novel pests, a strategic shift toward native and drought-adapted woody species is imperative. A local native species pool should be promoted for both public and private plantings. These species are evolutionarily suited to local conditions, typically require less irrigation water, and support native biodiversity, thereby enhancing the long-term sustainability and adaptive capacity of the urban ecosystem.
3. Promote the use of non-tree woody plants: Urban planners and managers must recognize that shrubs and vines are critical components of the green infrastructure. These woody plant forms provide essential ecosystem services—such as thermal regulation, habitat, and aesthetic value—while enhancing structural heterogeneity, biodiversity, and overall ecological resilience. Actively incorporating them into planting schemes, particularly in low-income areas where canopy cover is low and there are scarce shrubs and vines, can rapidly augment green cover and its associated benefits.
4. Empower communities through capacity building and stewardship: In low-income neighborhoods, resident engagement is a powerful tool for expanding and sustaining green cover on private and eligible communal land. We recommend establishing community-based training and certification programs, overseen by local municipalities. These programs would equip interested residents with the skills to select appropriate species for the site, plant, and care for woody vegetation in their own private spaces (e.g., front yards, interior gardens) and in authorized communal areas. By focusing on private stewardship and providing official recognition, these initiatives can foster a culture of care, legally amplify greening efforts beyond public rights-of-way, and build social resilience alongside ecological benefits.

Together, these recommendations should be considered as part of a strategy to transform Santiago’s streetscapes into more equitable, biodiverse, and climate-resilient urban forests.

## 4. Materials and Methods

### 4.1. Study Area

**Figure 4:**
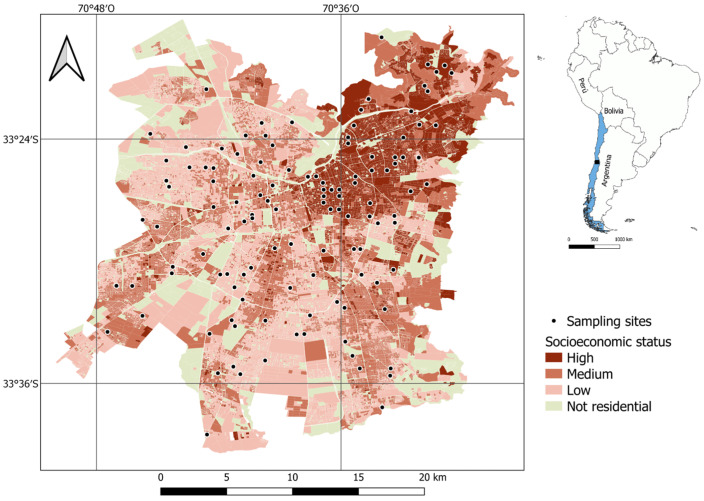
Sampling sites and socioeconomic levels in Santiago de Chile.

The research was conducted in Santiago, the capital of Chile, located at coordinates 33°26′ S and 70°39′ W, in central Chile (Figure 4). This city spans more than 650 km^2^ and is home to over 7 million residents (40% of the national population), making it Chile’s most populous city [71]. Santiago has a Mediterranean climate characterized by hot, dry summers (December to March) and cool, rainy winters (June to August) [72]. The warmest average temperature in the period 1991–2024 was around 23.3 °C, while the coldest temperature in winter exhibited an average of approximately 8.8 °C. The annual precipitation in the period 1991–2020 averaged about 286.2 mm [73].

Santiago is a city characterized by significant social segregation and an uneven distribution of green spaces. Higher-income residents predominantly inhabit the northeastern section of the city, which also boasts the most extensive vegetation coverage. Indeed, neighborhoods populated by individuals with greater financial means tend to feature larger green spaces, a higher density of trees, and a broader diversity of plant species compared to those inhabited by lower-income communities [32,74].

Santiago’s urban forest is diverse but exhibits a widespread use of introduced ornamental species for landscaping. At the city level, previous research has found that introduced species dominate the urban forest, with more than 80% of vascular flora and trees being non-native species [32,75]. In urban parks, introduced plant species are also widespread [75], accounting for approximately 70% of the park’s trees [39]. Several of these introduced plants originate from temperate regions with higher precipitation than central Chile, which entails high irrigation costs, especially during the dry seasons (spring and summer) [41].

### 4.2. Selection of Sampling Sites

In our research, we sampled 120 sites located in residential areas of varying socioeconomic statuses. These sites were identified by Villaseñor & Escobar [76], who used a stratified random selection design based on neighborhood socioeconomic level (obtained from https://observatoriodeciudades.com/ (accessed on 2021)) and distance to the city’s limit to select sampling sites. Neighborhood socioeconomic status was a categorical variable with three levels: high, medium, and low. High socioeconomic neighborhoods were mainly comprised by affluent individuals, typically university-educated, with an average annual household income exceeding USD 28,800. Medium socioeconomic neighborhoods were mainly comprised by households with technical or secondary education and a mean annual income surpassing USD 13,200. Conversely, low socioeconomic neighborhoods were dominated by households with less formal education, averaging an annual income below USD 8400 [77]. Stratifying by distance to the city’s limit ensured that all socioeconomic levels had a similar proportion of sample sites at the city’s edge (less than 5 km from the city limit) and in its interior (more than 5 km from the city limit) [76].

### 4.3. Woody Plants Surveys

Sampling took place between September and October 2022, the reproductive season of most plants in the Southern Hemisphere. Our surveys focused on the woody plants (trees, shrubs and vines) present in the residential streetscape, including public areas (e.g., streets, verges and medians) and street-visible private areas (e.g., front gardens). Thus, in each selected site, we established an 11 m radius circular plot (380 m^2^) where all visible woody species were identified. These plots have been successfully used to investigate urban forest composition and structure in our city (e.g., [32,39,74]). Trees with a diameter at breast height (DBH) greater than 2.5 cm were measured for height (using an altimeter Haga^©^, Gothenburg, Germany), diameter at breast high (DBH using a tree caliper Haglof^©^, Stockholm, Sweden), and crown diameter in two directions (N–S and W–E, using a forestry measuring tape Richter^©^, Düren, Germany). Other woody plants taller than 1.5 m were also measured. Crown size was used to calculate the crown area (in m^2^, using the formula for the area of an ellipse: Area = π × diameter_N-S_/2 × diameter_W-E_/2) [39]. The origin of plants (introduced or native) was determined based on the literature [78,79] and the web page World Flora Online [80].

### 4.4. Statistical Analysis

Initially, we calculated six response variables for each plot: species richness of woody plants, species richness of native woody plants, species richness of non-native woody plants, abundance of woody plants, abundance of native woody plants, and abundance of non-native woody plants. To assess differences in abundance and diversity across socioeconomic groups, we employed Generalized Linear Models (GLMs) with a Poisson distribution in R software version 4.1.2 [81]. Six distinct models were developed, each focusing on one of the six response variables calculated per plot. All models included socioeconomic status as a fixed effect (categorical variable with three levels: high, medium, low). We assessed models for overdispersion by calculating the sum of the squares of the Pearson residuals and comparing it to the model’s residual degrees of freedom through chi-square tests. Given that all abundance models were overdispersed, we included sampling plots as a random effect (*n* = 120) using Generalized Linear Mixed Models (GLMMs) in the “lme4” package [82].

Subsequently, we compared the height (cm) and crown area (m^2^) of woody plants across socioeconomic groups using a Linear Mixed Effects Model (LMM). Each model included socioeconomic level as a fixed effect and sampling plot as a random effect (n = 120). Height was fitted with a log-normal distribution, whereas crown area was fitted with a normal distribution. Then, we interpreted the effect of socioeconomic level in each model and visualized the results using the “effects” package [83]. Additionally, we calculated the proportion of woody vegetation type between trees and non-trees (shrubs, vines) by socioeconomic level and applied a chi-square test to establish significant differences.

To test the hypothesis that species composition differs significantly across different socioeconomic classes, we employed a permutational multivariate analysis of variance using the adonis function within the “vegan” package [84]. The analysis was based on a community dissimilarity matrix calculated using the Bray–Curtis distance measure. We used 200 free permutations to calculate the statistical significance of the F-statistic for the main effect of socioeconomics on the species matrix. To identify species that significantly drive compositional differences between socioeconomic groups, we used the SIMPER (Similarity Percentage) analysis using the “vegan” package [84]. This analysis shows species contributions to dissimilarity between residential streetscapes of different socioeconomic levels. SIMPER performs pairwise comparisons of groups of sampling units and finds the average contributions of each species to the average overall Bray–Curtis dissimilarity [85]. It also tests the probability of getting a larger or equal average contribution in a random permutation of the group factor (999 permutations). For both adonis and SIMPER analysis, we included species with more than five individuals. None of the undetermined taxa had more than five individuals (so they were excluded from analyses). These tests were performed in R software [81].

## 5. Conclusions

We found evident disparities in woody plant diversity, abundance, structure, and composition in residential streetscapes of different socioeconomic levels in Santiago de Chile, a Mediterranean-climate city located in Latin America. Residential streetscapes in wealthier zones exhibit greater woody plant diversity, abundance, and size than those in poorer zones, resulting in disparities in ecosystem services for citizens living in the same city. In addition, our study showed that the percentage of native woody plants in residential streetscapes (9.7%) was even lower than that reported at the city level and in urban parks, and raises concerns given the irrigation needed to sustain the massive use of introduced plants from climates with higher precipitation. The low proportion of woody native species compared to introduced species highlights the importance of promoting the use of native plants from the local region, which is currently experiencing drier conditions due to climate change.

The inclusion of non-tree woody plants in our analysis helped to verify that the luxury effect encompasses more than just urban trees. This phenomenon also affects smaller plants, which are less prevalent in areas with low socioeconomic levels. This disparity increases the lack of access to the ecosystem services these plants can provide. The challenge for the future greening of streetscapes is to include native (or to a lesser extent, introduced) shrubs and vines in the streets and front gardens of Santiago.

To reduce these inequalities and achieve a more environmentally just city, we suggest improving governance and equity-based investment, prioritizing local native species, promoting the use of non-tree woody plants, and empowering communities through capacity building and stewardship.

## Figures and Tables

**Table 1:** Summary of the number of woody plants and their origin recorded in residential streetscapes of different socioeconomic levels in Santiago de Chile.

	Total	Socioeconomic Level		
		Low	Medium	High
No. of plots	120	41	43	36
No. of plots with woody plants	118	40	43	35
No. of woody species	108	48	67	60
No. native of woody species	14 (13%)	5	7	7
No. introduced of woody species	94 (87%)	43	60	53
No. of woody plant individuals	557	160	196	201
No. of native woody plant individuals	54 (9.7%)	19	17	18
No. of introduced woody plant individuals	503 (90.3%)	183	179	141
No. of woody plant individuals/plot (mean ± SE)	4.64 ± 0.27	3.9 ± 0.39	4.56 ± 0.44	5.58 ± 0.5

**Table 2:** Results from generalized linear models (log-link) predicting species richness and generalized linear mixed models (log-link) predicting the abundance of total, native and introduced woody plants per plot according to socioeconomic level in residential areas of Santiago de Chile. Significance levels: *** *p* < 0.001; ** *p* < 0.01; * *p* < 0.05.

Response Variable		Coefficient	Std. Error	z Value	*p*-Value	
Species richness	Intercept	1.33	0.085	15.50	<0.001	***
	Socioeconomic-Medium	−0.12	0.119	−1.01	0.31	
	Socioeconomic-Low	−0.32	0.128	−2.54	0.01	*
Species richness of introduced woody plants	Intercept	1.23	0.090	13.62	<0.001	***
	Socioeconomic-Medium	−0.10	0.125	−0.79	0.43	
	Socioeconomic-Low	−0.35	0.135	−2.57	0.01	*
Species richness of native woody plants	Intercept	−1.02	0.28	−3.67	<0.001	***
	Socioeconomic-Medium	−0.34	0.41	−0.84	0.40	
	Socioeconomic-Low	−0.13	0.39	−0.33	0.74	
Total abundance	Intercept	1.65	0.10	16.882	<0.001	***
	Socioeconomic-Medium	−0.20	0.13	−1.529	0.13	
	Socioeconomic-Low	−0.36	0.14	−2.584	0.01	**
Abundance of introduced woody plants	Intercept	1.55	0.10	15.03	<0.001	***
	Socioeconomic-Medium	−0.20	0.14	−1.42	0.16	
	Socioeconomic-Low	−0.39	0.15	−2.67	0.01	**
Abundance of native woody plants	Intercept	−1.55	0.45	−3.42	<0.001	***
	Socioeconomic-Medium	−0.6	0.46	−0.86	0.39	
	Socioeconomic-Low	−0.13	0.13	−0.26	0.80	

**Table 3:** Results from linear mixed effects models predicting crown area and height of woody plants according to socioeconomic level in residential areas of Santiago de Chile. Significance levels: *** *p* < 0.001; * *p* < 0.05.

Response Variables		Coefficient	Std. Error	t-Value	*p*-Value	
Crown area	Intercept	6.71	0.97	6.910	<0.001	***
	Socioeconomic-Medium	−3.05	1.36	−2.249	0.03	*
	Socioeconomic-Low	−3.04	1.42	−2.150	0.03	*
Height (log-normal)	Intercept	6.04	0.05	114.344	<0.001	***
	Socioeconomic-Medium	−0.29	0.07	−3.934	<0.001	***
	Socioeconomic-Low	−0.19	0.08	−2.479	0.015	*

**Table 4:** Results from SIMPER analysis showing species contributions to dissimilarities between socioeconomic groups. Significance levels: *** *p* < 0.001; * *p* < 0.05; *p* < 0.1.

Species	Origin	Average Abundance in Socioeconomic Groups			Average Dissimilarities (%)					
		High	Medium	Low	High vs. Low		High vs. Medium		Low vs. Medium	
*Liquidambar styraciflua*	Introduced	0.79	0.20	0.03	0.11	*	0.12	***	0.04	
*Robinia pseudoacacia*	Introduced	0.46	0.23	0.79	0.14	*	0.09		0.13	
*Platanus* x *hispanica*	Introduced	0.42	0.26	0.03	0.07		0.09	*	0.05	
*Pittosporum tobira*	Introduced	0.30	0.11	0.03	0.04		0.04	*	0.01	
*Ligustrum lucidum*	Introduced	0.21	0.51	0.38	0.06		0.08		0.09	
*Fraxinus excelsior*	Introduced	0.18	0.06	0.18	0.05		0.04		0.04	
*Melia azedarach*	Introduced	0.18	0.20	0.15	0.04		0.04		0.05	
*Quillaja saponaria*	Native	0.18	0.29	0.12	0.04		0.06		0.06	
*Ligustrum sinense*	Introduced	0.18	0.17	0.09	0.03		0.04		0.03	
*Acer negundo*	Introduced	0.12	0.23	0.44	0.07		0.05		0.08	.
*Schinus areira*	Native	0.12	0.03	0.32	0.05	.	0.02		0.05	
*Jacaranda mimosifolia*	Introduced	0.12	0.11	0.09	0.03		0.03		0.02	
*Prunus cerasifera f. nigra*	Introduced	0.09	0.14	0.12	0.03		0.03		0.04	
*Bougainvillea* sp.	Introduced	0.09	0.11	0.03	0.02		0.03		0.02	
*Punica granatum*	Introduced	0.09	0.09	0.03	0.02		0.03		0.02	
*Pyracantha coccinea*	Introduced	0.06	0.26	0.21	0.03		0.04		0.05	
*Prunus cerasifera*	Introduced	0.03	0.20	0.15	0.03		0.02		0.04	*
*Prunus* sp.	Introduced	0.03	0.09	0.06	0.01		0.02		0.02	
*Citrus limon*	Introduced	0.00	0.20	0.12	0.03		0.04		0.05	*
*Ailanthus altissima*	Introduced	0.00	0.23	0.09	0.01		0.03		0.04	*

## Data Availability

The data presented in this study are available on request from the corresponding author. The data are not publicly available because they have not yet been curated for deposition in a public repository.

## Appendix A

**Table A1:** Woody plants from Santiago. Nomenclature followed The Plant List (2013), and the taxonomical information according to the APG IV system, origin in Chile (N: native species; I: introduced species).

Order	Family	Genus	Species	Origin
Pinopsida	Araucariaceae	*Araucaria*	*araucana*	N
Pinopsida	Cupressaceae	*Cupressus*	*macrocarpa*	I
Pinopsida	Cupressaceae	*Cupressus*	sp.	I
Pinopsida	Cupressaceae	*Thuja*	sp.	I
Pinopsida	Pinaceae	*Cedrus*	sp.	I
Pinopsida	Pinaceae	*Pinus*	*pinaster*	I
Magnoliopsida	Adoxaceae	*Viburnum*	*odoratissimum*	I
Magnoliopsida	Adoxaceae	*Viburnum*	*tinus*	I
Magnoliopsida	Adoxaceae	*Viburnum*	sp.	I
Magnoliopsida	Altingiaceae	*Liquidambar*	*styraciflua*	I
Magnoliopsida	Anacardiaceae	*Schinus*	*areira*	N
Magnoliopsida	Apocynaceae	*Nerium*	*oleander*	I
Magnoliopsida	Araliaceae	*Hedera*	sp.	I
Magnoliopsida	Araliaceae	*Schefflera*	*arboricola*	I
Magnoliopsida	Bignoniaceae	*Catalpa*	*bignonioides*	I
Magnoliopsida	Bignoniaceae	*Jacaranda*	*mimosifolia*	I
Magnoliopsida	Bignoniaceae	*Tecoma*	*stans*	I
Magnoliopsida	Cannabaceae	*Celtis*	*australis*	I
Magnoliopsida	Caprifoliaceae	*Abelia*	*grandiflora*	I
Magnoliopsida	Caprifoliaceae	*Abelia*	sp.	I
Magnoliopsida	Celastraceae	*Euonymus*	*japonicus*	I
Magnoliopsida	Celastraceae	*Maytenus*	*boaria*	N
Magnoliopsida	Elaeocarpaceae	*Aristotelia*	*chilensis*	N
Magnoliopsida	Elaeagnaceae	*Elaeagnus*	*angustifolia*	I
Magnoliopsida	Ericaceae	*Arbutus*	*unedo*	I
Magnoliopsida	Euphorbiaceae	*Euphorbia*	sp.	I
Magnoliopsida	Fabaceae	*Acacia*	*dealbata*	I
Magnoliopsida	Fabaceae	*Acacia*	*karroo*	I
Magnoliopsida	Fabaceae	*Acacia*	sp.	I
Magnoliopsida	Fabaceae	*Bauhinia*	*forficata*	I
Magnoliopsida	Fabaceae	*Erythrina*	*crista-galli*	I
Magnoliopsida	Fabaceae	*Erythrostemon*	*gilliesii*	I
Magnoliopsida	Fabaceae	*Parkinsonia*	*aculeata*	I
Magnoliopsida	Fabaceae	*Prosopis*	*chilensis*	N
Magnoliopsida	Fabaceae	*Robinia*	*pseudoacacia*	I
Magnoliopsida	Fabaceae	*Styphnolobium*	*japonicum*	I
Magnoliopsida	Fabaceae	*Tara*	*spinosa*	N
Magnoliopsida	Fabaceae	*Vachellia*	*caven*	N
Magnoliopsida	Fagaceae	*Quercus*	*falcata*	I
Magnoliopsida	Fagaceae	*Quercus*	*robur*	I
Magnoliopsida	Juglandaceae	*Juglans*	*regia*	I
Magnoliopsida	Lamiaceae	*Salvia*	*rosmarinus*	I
Magnoliopsida	Lauraceae	*Cryptocarya*	*alba*	N
Magnoliopsida	Lauraceae	*Laurus*	*nobilis*	I
Magnoliopsida	Lauraceae	*Persea*	*americana*	I
Magnoliopsida	Lauraceae	*Persea*	sp.	I
Magnoliopsida	Loranthaceae	*Tristerix*	sp.	N
Magnoliopsida	Lythraceae	*Lagerstroemia*	*indica*	I
Magnoliopsida	Lythraceae	*Punica*	*granatum*	I
Magnoliopsida	Magnoliaceae	*Liriodendron*	*tulipifera*	I
Magnoliopsida	Magnoliaceae	*Magnolia*	*soulangeana*	I
Magnoliopsida	Magnoliaceae	*Magnolia*	*grandiflora*	I
Magnoliopsida	Malvaceae	*Brachychiton*	*acerifolius*	I
Magnoliopsida	Malvaceae	*Brachychiton*	*populneus*	I
Magnoliopsida	Malvaceae	*Hibiscus*	*boryanus*	I
Magnoliopsida	Malvaceae	*Tilia*	*americana*	I
Magnoliopsida	Meliaceae	*Melia*	*azedarach*	I
Magnoliopsida	Moraceae	*Ficus*	*benjamina*	I
Magnoliopsida	Moraceae	*Ficus*	*elastica*	I
Magnoliopsida	Moraceae	*Morus*	*alba*	I
Magnoliopsida	Myrtaceae	*Eucalyptus*	sp.	I
Magnoliopsida	Myrtaceae	*Myrtus*	*communis*	I
Magnoliopsida	Nyctaginaceae	*Bougainvillea*	sp.	I
Magnoliopsida	Oleaceae	*Fraxinus*	*excelsior*	I
Magnoliopsida	Oleaceae	*Jasminum*	*mesnyi*	I
Magnoliopsida	Oleaceae	*Ligustrum*	*lucidum*	I
Magnoliopsida	Oleaceae	*Ligustrum*	*sinense*	I
Magnoliopsida	Oleaceae	*Olea*	*europaea*	I
Magnoliopsida	Pittosporaceae	*Pittosporum*	*tenuifolium*	I
Magnoliopsida	Pittosporaceae	*Pittosporum*	*tobira*	I
Magnoliopsida	Pittosporaceae	*Pittosporum*	*undulatum*	I
Magnoliopsida	Platanaceae	*Platanus*	*hispanica*	I
Magnoliopsida	Proteaceae	*Grevillea*	*robusta*	I
Magnoliopsida	Quillajaceae	*Quillaja*	*saponaria*	N
Magnoliopsida	Rosaceae	*Cotoneaster*	*coriaceus*	I
Magnoliopsida	Rosaceae	*Cotoneaster*	sp.	I
Magnoliopsida	Rosaceae	*Eriobotrya*	*japonica*	I
Magnoliopsida	Rosaceae	*Malus*	*purpurea*	I
Magnoliopsida	Rosaceae	*Prunus*	*armeniaca*	I
Magnoliopsida	Rosaceae	*Prunus*	*cerasifera*	I
Magnoliopsida	Rosaceae	*Prunus*	*cerasifera f. nigra*	I
Magnoliopsida	Rosaceae	*Prunus*	*dulcis*	I
Magnoliopsida	Rosaceae	*Prunus*	*persica*	I
Magnoliopsida	Rosaceae	*Prunus*	sp.	I
Magnoliopsida	Rosaceae	*Pyracantha*	*coccinea*	I
Magnoliopsida	Rosaceae	*Rosa*	sp.	I
Magnoliopsida	Rubiaceae	*Coprosma*	*baueri*	I
Magnoliopsida	Rutaceae	*Citrus*	*sinensis*	I
Magnoliopsida	Rutaceae	*Citrus*	*reticulata*	I
Magnoliopsida	Rutaceae	*Citrus*	*limon*	I
Magnoliopsida	Salicaceae	*Populus*	*alba*	I
Magnoliopsida	Salicaceae	*Populus*	*deltoides*	I
Magnoliopsida	Salicaceae	*Populus*	sp.	I
Magnoliopsida	Sapindaceae	*Acer*	*japonicum*	I
Magnoliopsida	Sapindaceae	*Acer*	*negundo*	I
Magnoliopsida	Sapindaceae	*Acer*	*pseudoplatanus*	I
Magnoliopsida	Scrophulariaceae	*Buddleja*	*globosa*	N
Magnoliopsida	Scrophulariaceae	*Myoporum*	*laetum*	I
Magnoliopsida	Simaroubaceae	*Ailanthus*	*altissima*	I
Magnoliopsida	Solanaceae	*Brugmansia*	*arborea*	N
Magnoliopsida	Solanaceae	*Brunfelsia*	*pauciflora*	I
Magnoliopsida	Solanaceae	*Cestrum*	*parqui*	N
Magnoliopsida	Theaceae	*Camellia*	sp.	I
Magnoliopsida	Ulmaceae	*Ulmus*	*minor*	I
Magnoliopsida	Winteraceae	*Drimys*	*winteri*	N
Magnoliopsida	Unidentified	*-*	*Morphotype-tree-01*	I
Magnoliopsida	Unidentified	*-*	*Morphotype-shrub-01*	I
Magnoliopsida	Unidentified	*-*	*Morphotype-climbing-01*	I
